# Prevalence and association of elevated total serum IgE levels with ICU admissions and poor asthma control: a retrospective study in a tertiary hospital in Saudi Arabia

**DOI:** 10.1186/s13223-025-01003-z

**Published:** 2025-11-28

**Authors:** Hassan Alwafi, Husna Irfan Thalib, Ayesha Jamal, Safaa M. Alsanosi, Abdallah Y. Naser, Abdulelah M. Aldhahir, Abdullah A. Alqarni, Jaber S. Alqahtani, Saeed M. Alghamdi, Mohammad Saleh Dairi, Omaima Ibrahim Badr

**Affiliations:** 1 Department of Pharmacology and Toxicology, College of Medicine, Umm Al- Qura University, Makkah, Saudi Arabia; 2 Batterjee Medical College, Jeddah, Saudi Arabia; 3 Department of Applied Pharmaceutical Sciences and Clinical Pharmacy, Faculty of Pharmacy, Isra University, Amman, Jordan; 4 Respiratory Therapy Program, Department of Nursing, College of Nursing and Health Sciences, Jazan University, Jazan University, Jazan, Saudi Arabia; 5 Department of Respiratory Therapy, Faculty of Medical Rehabilitation Sciences, King Abdulaziz University, Jeddah, Saudi Arabia; 6 Respiratory Therapy Unit, King Abdulaziz University Hospital,, Jeddah, Kingdom of Saudi Arabia; 7 Department of Respiratory Care, Prince Sultan Military College of Health Sciences, 34313 Dammam, Saudi Arabia; 8 Respiratory Care Program, Clinical Technology Department, Faculty of Applied Medical Sciences, Umm Al-Qura University, 24382 Makkah, Saudi Arabia; 9 Department of Medicine, College of Medicine, Umm Al-Qura University, Makkah, Saudi Arabia; 10 Department of Chest Medicine, Faculty of Medicine, Mansoura University, Mansoura, 35516 Egypt; 11 Department of Pulmonary Medicine, Al Noor Specialist Hospital, 20424 Mecca, Saudi Arabia

**Keywords:** Asthma, Immunoglobulin E, ICU admission, Retrospective study

## Abstract

**Objective:**

This study aimed to determine the prevalence of elevated serum Immunoglobulin E (IgE) levels among adult patients with asthma and to investigate the association between IgE levels and hospital admissions, ICU admissions, asthma control, and other clinical outcomes.

**Methods:**

This was a retrospective single-centre study. Adult patients with a confirmed diagnosis of asthma between 28 February 2023 and 1 March 2024 at Al-Noor Specialist Hospital, Mecca, Saudi Arabia, were included. Patients were excluded if their records lacked spirometry or serum IgE data. Population characteristics were presented as frequency for categorical variables and median with interquartile range for continuous variables. Logistic regression analysis was used to identify predictors of severe asthma outcomes stratified by IgE levels.

**Results:**

The study included 807 asthma patients, with a majority being females (68.9%) and a mean age of 48.2 years. A total of 311 patients (38.5%) had elevated serum IgE levels (> 100 IU). Patients with high IgE levels had significantly higher rates of hospital admission due to asthma (16.4% vs. 7.1%, *p* < 0.001) and ICU admission (4.2% vs. 1.2%, *p* = 0.008). They were also less likely to have controlled asthma (42.4% vs. 78.9%, *p* < 0.001) and more likely to report frequent acute care service visits (62.1% vs. 32.5%, *p* < 0.001). Logistic regression showed that elevated IgE levels were significantly associated with increased odds of hospital admission (OR = 2.6, 95% CI = [1.6–4.0], *p* < 0.001), ICU admission (OR = 3.5, 95% CI = [1.3–9.4], *p* = 0.011), uncontrolled asthma (OR = 0.2, 95% CI = [0.1–0.3], *p* < 0.001), and frequent ACS visits (OR = 0.3, 95% CI = [0.2–0.4], *p* < 0.001).

**Conclusions:**

Elevated serum IgE levels are significantly associated with poorer asthma control, increased hospital admissions, and higher ICU admission rates. Stratifying asthma patients based on IgE levels may help identify high-risk individuals and guide management strategies through a targeted approach.

## Introduction

Asthma is a chronic inflammatory airway disease characterized by intermittent wheezing, shortness of breath, chest tightness, and cough. Intensity and frequency of the symptoms are variable as it can range from mild to severe and potentially life-threatening. Common precipitants of asthma attacks include allergens such as pollen and cat dander, infection of the respiratory tract, exercise, cold air, and emotional stress. The chronic inflammatory condition occurs as a result of interaction between genetic predisposition and environmental exposures that collectively trigger an excessive immune response. The exaggerated immune responses results in inflammation, thickening of the bronchial walls, and excess mucus secretion, which obstructs air passage [1].

Asthma control and prevention of acute attack are the focus of management. Rapid relief is provided by short-acting beta-agonists and long-term by inhaled corticosteroids or, if necessary, long-acting beta-agonists. Elimination of known allergens is also required. Follow-up with a physician on a regular basis encourages compliance with treatment and allows timely adjustment of the treatment plan [2]. Asthma remains a global health problem that is responsible for a lot of illness and healthcare costs despite progress in diagnosis and treatment [3].

Immunoglobulin E (IgE) is the central mediator of atopic asthma. After exposure to environmental allergens, B cells release IgE antibodies that attach to high-affinity FcεRI receptors on mast cells and basophils. In return contact with the same allergen, the attached IgE molecules cross-link and trigger the release of inflammatory mediators such as histamine, leukotrienes, and cytokines. The resulting airway inflammation, narrowing, edema, and mucus overproduction constitute the typical asthma features [4].

In the more serious forms of the disease, serum IgE levels are often elevated, in line with chronic atopic sensitization. These individuals have heightened airway responsiveness, greater exacerbations, and a reduced response to standard treatment. IgE thereby became a prime target for therapy. Omalizumab, a monoclonal antibody directed against circulating IgE, prevents its binding to basophils and mast cells and has been shown to reduce exacerbations, hospital admissions, and improve asthma control as a whole [5].

In Saudi Arabia, asthma affects approximately 11.3% of adults with great regional variation and additional burden on the health system [6]. Studies from across the world have indicated that there is a correlation between high serum IgE and increased severity of asthma [7–9]. However, the extent to which serum IgE will correlate with severe episodes such as admission to an ICU is not known. Though in children results have been strongly correlated with IgE and disease severity, the results in adults are different [7, 9].

Severe asthma remains challenging to treat. Many patients continue to have exacerbations while they are on high-dose inhaled corticosteroids and long-acting bronchodilators. Biologic treatments, including anti-IgE and anti-IL-5 therapies, have now extended the treatment options for this population [10]. To the best of our knowledge, no Saudi Arabian study has been carried out to investigate the relationship between serum IgE concentrations and ICU admissions for asthma exacerbations. Investigation of this correlation may allow for the identification of patients at increased risk and facilitate more tailored treatment strategies.

## Methods

### Study design and study settings

This was a retrospective single-centre cross-sectional descriptive study. Data were collected between 28 February 2023 and 1 March 2024 from Al-Noor Specialist Hospital, a tertiary centre in Mecca, Saudi Arabia. Details of the data collection method and study settings were previously described [11]. The study included adult patients aged ≥ 18 years with a confirmed clinical and functional diagnosis of asthma (increased post bronchodilator FEV1 from baseline in by ≥ 12% and ≥ 200 mL and decreased FEV1/FVC ≤ 70%) documented in the hospital’s medical records. Only patients with documented spirometry findings and serum Immunoglobulin E (IgE) levels were included. Clinical assessment, asthma management, and comorbid conditions were addressed by multidisciplinary teams led by qualified consultants across internal medicine, pulmonology, and allergy specialties.

### Study variables

A standardized data collection sheet was used to retrieve information from patients’ electronic medical records. Demographic data included age, sex, body mass index (BMI), and smoking status. Comorbidities were documented based on clinical history and chart review and included variables such as allergic dermatitis, atopic dermatitis, gastritis/ gastroesophageal reflux disease (GERD), sinusitis, diabetes mellitus, hypertension, and others, as per the Charlson comorbidity index classification. Allergic dermatitis and atopic dermatitis were retained as distinct diagnostic categories. Atopic dermatitis denotes a chronic, relapsing eczematous disorder associated with atopy, elevated serum IgE, and a personal or family history of allergic disease. In contrast, allergic dermatitis refers to allergic contact or other hypersensitivity-mediated cutaneous reactions arising from direct allergen exposure in non-atopic individuals. Data including asthma medications (e.g., inhaled corticosteroids [ICS], long-acting beta-agonists [LABA], leukotriene receptor antagonists, biologics like omalizumab), history of asthma exacerbations, hospitalization, and ICU admissions were collected. Polypharmacy was defined as the use of five or more medications in a concurrent manner, including asthma-related treatments.

Lung function parameters including forced vital capacity (FVC), forced expiratory volume in 1 s (FEV₁), FEV₁/FVC ratio, and peak expiratory flow (PEF), were also recorded. Serum laboratory data, including total IgE levels and vitamin D, were retrieved from the most recent results available in the hospital’s electronic system. Total serum IgE levels were measured using a standardized immunoassay method (ImmunoCAP, Thermo Fisher Scientific). Values were obtained within 12 months prior to or during the index clinical encounter, and a cut-off value of 100 IU/mL was used to define elevated IgE levels, consistent with commonly accepted laboratory reference ranges. Asthma control was assessed using the Asthma Control Test (ACT) scores, which were interpreted according to the original validation criteria [12]; a score of < 20 was considered indicative of suboptimal asthma control, while scores ≥ 20 represented controlled asthma. Acute care service visits for asthma were also recorded in the past 2 years. Data on skin-prick testing or allergen-specific IgE were not routinely available for all patients, and therefore atopic status could not be included in the analysis.

### Statistical analysis

Data were analyzed using Statistical Package for Social Science (SPSS) software, version 27 (IBM Corp, Armonk, NY, USA). Categorical variables were summarized using frequencies and percentages, while continuous variables were presented as mean with standard deviation (SD) or median with interquartile range (IQR), as appropriate. Between-group comparisons were performed using the Mann–Whitney U test for continuous variables and chi-square test for categorical variables. Binary logistic regression analysis was conducted to evaluate the association between high IgE levels and selected clinical outcomes, including hospital admissions, ICU admissions, asthma control status, and frequency of ACS visits. Statistical significance was considered for *P* < 0.05. 95% confidence intervals (CIs) were reported for odds ratios (ORs).

## Ethical approval and consent to participate

This study was approved by the institutional ethics board at the Ministry of Health in Saudi Arabia (No. H-02-K-076–0523–951). Patients were ensured that their clinical data may be used for research purposes only. The requirement for written informed consent was waived by the ethics committee due to the retrospective nature of the study. All procedures were conducted in accordance with the ethical standards of the Declaration of Helsinki.

## Results

### Patients’ baseline characteristics

A total of 807 asthma patients were included in the study. The mean age was 48.2 ± 16.0 years, and the majority of the participants were females (68.9%). The mean body mass index (BMI) was 29.1 ± 6.3 kg/m², and 15.0% of the patients were current smokers. A family history of asthma was reported in 24.2% of the cohort. Several comorbidities were frequently observed, with the most common being allergic dermatitis (38.7%), gastritis or GERD (29.9%), diabetes mellitus (20.3%), and hypertension (19.6%). A total of 440 patients (54.5%) were on polypharmacy. Other commonly reported conditions included atopic dermatitis, sinusitis, osteoporosis or osteopenia, obstructive sleep apnea (OSA), ischemic heart disease, psychiatric disorders, and various less common conditions including malignancy and connective tissue diseases. A detailed summary of demographic and clinical characteristics is presented in Table 1.

**Table 1:** Patients’ baseline characteristics

Variable		Frequency		Percentage		
Age: Mean (SD) years		48.2 (16.0) years				
*Sex*						
Females		556		68.9%		
BMI: Mean (SD) kg/cm^2^		29.1 (6.3) kg/cm^2^				
Smoking status (current smoker)		121		15.0%		
Family history of asthma		195		24.2%		
Food allergy		175		21.7%		
Drug allergy		16		2.0%		
Atopic dermatitis		101		12.5%		
Contact dermatitis		312		38.7%		
Sinusitis		99		12.3%		
Gastritis/Gastroesophageal Reflux Disease (GERD)		241		29.9%		
Osteoporosis/ Osteopenia		18		2.2%		
Obstructive Sleep Apnea (OSA)		50		6.2%		
Hypertension		158		19.6%		
Diabetes mellitus		164		20.3%		
Ischemic heart disease		64		7.9%		
Heart failure		16		2.0%		
Chronic kidney disease		6		0.7%		
Rheumatological disease		3		0.4%		
Stroke		3		0.4%		
Dementia		2		0.2%		
Peripheral vascular diseases		1		0.1%		
Connective tissue diseases		14		1.7%		
Tuberculosis history		1		0.1%		
Tumor/malignancy		2		0.2%		
Metastasis		1		0.1%		
Lymphoma		1		0.1%		
Psychiatric disorders		56		6.9%		
Polypharmacy (5 or more)		440		54.5%		
Oral hypoglycemic/insulin		161		20.0%		
Anti-platelet		79		9.8%		
Anti-coagulants		13		1.6%		
Anti-hypertensive		171		21.2%		
Heart Failure (HF) medications		18		2.2%		
Anti hyperlipidemics		114		14.1%		
Anti-psychotics/depressant		56		6.9%		
Proton Pump Inhibitors (PPI)		253		31.4%		
Antihistaminic		283		35.1%		
Dugs for osteoporosis		23		2.9%		

### Asthma medication utilization profile

Inhaled corticosteroids (ICS) were the most commonly prescribed asthma medication and were used in 87.7% of patients. Long-acting beta-agonists (LABA) were prescribed in 86.2% of cases, followed by leukotriene receptor antagonists (Montelukast) in 42.2%, and long-acting muscarinic antagonists (LAMA) in 34.8%. Systemic corticosteroids were used in 16.4% of patients. Only a small proportion of patients received biologic therapies, with omalizumab prescribed in 2.7% and other biologics including benralizumab and dupilumab in less than 1%. The full medication profile is provided in Table 2.

**Table 2:** Asthma medications utilization

Variable	Frequency	Percentage
SABA	6	0.7%
LABA	696	86.2%
LAMA	281	34.8%
ICS	708	87.7%
Systemic corticosteroids	132	16.4%
Omalizumab	22	2.7%
Benralizumab	4	0.5%
dupilumab	1	0.1%
Other respiratory medications (e.g. montelukast)	341	42.2%

### IgE level and spirometry parameters

Among the total sample of 807 patients, 311 (38.5%) were identified as having elevated serum IgE levels. The median IgE level in the normal group was 37.0 IU/mL with an interquartile range (IQR) of 40.88, whereas in the high IgE group, the median was markedly higher at 418.0 IU/mL with an IQR of 618.0. Spirometry parameters, including actual values for FVC, FEV₁, FEF25–75%, and PEF, were compared between patients with normal and high IgE levels using the Mann–Whitney U test. The analysis showed no statistically significant differences in the median values of these spirometric measures across the two groups (*p* > 0.05 for all comparisons), indicating similar baseline pulmonary function. However, a statistically significant difference was observed in serum vitamin D levels. Patients with elevated IgE had significantly lower median vitamin D concentrations compared to those with normal IgE levels (*p* < 0.001). Detailed results of the lung function and laboratory findings stratified by IgE status are presented in Table 3.

**Table 3:** Spirometry parameters and lab tests

Variable	Median value for patients with normal IgE level (interquartile range)	Median value for patients with high IgE level (interquartile range)	*P*-value
Vitamin D	30.5 (27.2)	30.4 (27.3)	< 0.001
FVC ( L)	2.9 (1.2)	2.9 (1.2)	0.573
FVC (%)	81.0 (18)	82.0 (16)	0.606
FEV1 (L )	1.7 (1.0)	1.7 (1.0)	0.758
FEV1 (%) pre bronchodilator	59.0 (20)	60.0 (19)	0.854
FEV1 (%) post bronchodilator	74.0 (24)	76.0 (23)	0.562
FEF25-75 (L)	2.1 (1.3)	2.1 (1.2)	0.338
FEF25-75 (%)	68.0 (26)	69.0 (30.0)	0.513
PEF(L/sec)	5.0 (2.1)	5.1 (2.2)	0.771
PEF (%)	76.5 (23)	76.0 (22)	0.813

### Patients’ hospitalization profile stratified by IgE level

Patients with elevated IgE levels experienced worse clinical outcomes in terms of hospitalization and asthma control. Specifically, hospital admissions due to asthma were significantly more frequent in the elevated IgE group (16.4%) compared to the normal IgE group (7.1%) (*p* < 0.001). Similarly, ICU admissions in the past two years were more common among those with high IgE levels (4.2% vs. 1.2%, *p* = 0.008). The frequency of acute care service visits was higher among those with elevated IgE, with only 37.9% having fewer than 21 acute care service visits in the past 2 years compared to 67.5% in the normal IgE group (*p* < 0.001). Furthermore, asthma control, as measured by the Asthma Control Test (ACT), was also significantly poorer in patients with elevated IgE, with only 42.4% achieving controlled asthma compared to 78.9% in the normal IgE group (*p* < 0.001). These outcomes are summarized in Table 4.

**Table 4:** Patients’ hospitalization profile and asthma control stratified by IgE level

Variable	Patients with normal IgE level (n = 492)			Patients with high IgE level (n = 311)			*P*-value
	Frequency	Percentage		Frequency	Percentage		
Hospital admission due to Asthma	35	7.1%		51	16.4%		< 0.001
ICU admission last 2 years due to asthma	6	1.2%		13	4.2%		0.008**
Lower number of acute care service visits (below 21) last 2 years	332	67.5%		118	37.9%		< 0.001
*ACT*							
Controlled asthma (ACS ≥ 20)	388	78.9%		132	42.4%		< 0.001

### The association between high IgE level and patients’ outcomes

Binary logistic regression analysis showed that elevated serum IgE levels were significantly associated with worse asthma-related outcomes. Patients with high IgE were more likely to be admitted to the hospital due to asthma, with an odds ratio (OR) of 2.6 (95% confidence interval [CI]: 1.6–4.0, *p* < 0.001). The likelihood of ICU admission within the past two years was also significantly higher in this group (OR = 3.5, 95% CI: 1.3–9.4, *p* = 0.011). Additionally, patients with elevated IgE were significantly less likely to have controlled asthma based on ACT scores (OR = 0.2, 95% CI: 0.1–0.3, *p* < 0.001) and were also less likely to have fewer acute care service visits (OR = 0.3, 95% CI: 0.2–0.4, *p* < 0.001). These findings are detailed in Table 5.

**Table 5:** Binary logistic regression analysis

	Odds ratio (95% confidence interval)	*P*-value
Hospital admission due to Asthma	2.6 (1.6-4.0)	< 0.001
ICU admission last 2 years due to asthma	3.5 (1.3–9.4)	0.011*
Controlled Asthma (ACS ≥ 20)	0.2 (0.1–0.3)	< 0.001
Lower number of acute care service visits last 2 years	0.3 (0.2–0.4)	< 0.001

## Discussion

In this research, 807 asthma patients were examined for the relationship between the severity of the disease and the serum immunoglobulin E (IgE) level. The age range of the subjects was 48.2 years, and the subjects were mostly female (68.9%). Elevated IgE was present in 38.5% of the patients and was related to worse disease control and more unfavorable clinical outcomes. The patients with elevated IgE levels were hospitalized more frequently for asthma attacks (16.4%) and intensive care unit admissions (4.2%). They also exhibited lower Asthma Control Test (ACT) scores and experienced more severe attacks compared to the patients with normal IgE levels. The findings suggest that measurement of serum IgE may have a potential application for the detection of patients at risk of developing more severe asthma attacks.

The results of this study are in agreement with earlier findings. Aziz and others (2023) also performed similar research at Aga Khan University Hospital, Pakistan, and found that patients with raised levels of IgE had more exacerbations and required greater healthcare help than patients with normal levels. Their research also indicated an association of higher levels of IgE, positive family history of asthma, and higher healthcare utilization, especially among children [9].

James et al. (2019) also reported that IgE levels to allergens were higher during acute attacks in children than when asthma was best controlled, indicating that IgE measurement during an attack can be utilized in identifying the pertinent stimuli [13]. Sherenian (2015) found asthmatic children to have higher levels of serum IgE compared to non-asthmatic controls, and serum IgE was related to disease severity. That study, however, could not determine if increases in IgE had occurred before or at the same time as exacerbations [14]. On the other hand, Jackson et al. (2020) added that elevated IgE in isolation was not a predictor of future exacerbation, while elevated baseline eosinophil count was a better predictor of risk and reflected the multi-factorial pathophysiology of asthma [15].

Increased IgE’s association with elevated hospital or ICU admission rates could be explained by well-established immunopathological mechanisms [16]. Elevated IgE reflects sensitization to environmental allergens such as dust mites, pollen, or animal dander. Re-exposure to such allergens triggers basophils and mast cells through IgE-dependent mechanisms, leading to the release of histamine, leukotrienes, and cytokines. Airway hyperresponsiveness, mucus overproduction, and inflammation of the bronchi are all mediated by these mediators and result in airflow obstruction and worsening of asthma symptoms [16]. Individuals with more than one allergic illness, such as allergic rhinitis or atopic dermatitis, have more difficult-to-treat disease and a higher probability of developing severe exacerbations. Stress, worry, and other psychosocial issues may even complicate the symptoms further and compromise adherence to medication [17].

Since the relationship between elevated IgE and uncontrolled asthma is so potent, targeted treatment is especially prominent within this group as an important factor [18, 19]. Omalizumab is a free IgE-binding, antibody that blocks interaction with mast cell and basophil receptors and thereby reduces release of inflammatory mediators. Clinical experience has shown that omalizumab reduces exacerbation rate, hospitalization, and improves symptom control and quality of life [18]. Inhaled corticosteroids continue as the foundation of asthma management by reducing airway inflammation and hyperresponsiveness. Coupling these drugs with education and follow-up on a regular basis is critical to attain maximal control [19].

Longitudinal studies in the future must be used to assess the temporal association between serum IgE levels and asthma control. These studies could establish if elevation of IgE comes before exacerbations or merely represents chronic inflammation. There are several limitations to this study. Its cross-sectional design limits causal interpretation, and variation in laboratory technique of IgE measurement could have influenced outcomes. Systematically collected allergic comorbidities such as rhinitis and dermatitis were not obtained, nor was skin-prick testing or allergen-specific IgE performed, excluding full assessment of atopy status. These limitations need to be addressed in future studies to improve understanding of the pathophysiological role of IgE in asthma and outcomes.

Omalizumab, a recombinant humanized IgG1 monoclonal antibody directed against IgE, is now recommended as add-on therapy in those with severe asthma who remain uncontrolled on high-dose inhaled corticosteroids and long-acting bronchodilators. Benefit is generally seen within sixteen weeks of therapy. Response cannot be predicted beforehand of this interval, but IgE level monitoring during therapy is useful to ensure adequate dosing and therapeutic level [20, 21].

In short, based on this research, it is apparent that high serum IgE is heavily associated with inadequate asthma control. Those with higher levels of IgE had poorer symptom control, more hospitalizations, and higher intensive care unit admission risk. Monitoring IgE on a routine basis will enable clinicians to recognize at-risk patients and prescribe more tailored treatment regimens in an effort to minimize exacerbations and enhance quality of care.

## Conclusion

The study found a significant association between elevated serum IgE levels and worse asthma outcomes. Subjects with higher IgE more often experienced uncontrolled symptoms, hospitalizations, and intensive care treatments. Nearly two-thirds of the subjects had raised IgE, and those with elevated IgE also had more severe and frequent exacerbations. These findings suggest that elevated IgE may act as a marker of increased disease activity or allergen exposure rather than being directly causal. These findings suggest that regular monitoring of IgE levels may help in the treatment of asthma and identify those patients who are at increased risk of complication. Evaluation of disease severity and IgE levels might help in more targeted therapy and might open the way for the application of biologic therapy in some patients. Additional prospective studies are needed to evaluate the impact of IgE-targeted therapy on long-term asthma control and outcome.

## Data Availability

The datasets used and analyzed during the current study are available from the corresponding author upon reasonable request.
